# Seasonal variation, treatment outcome, and its associated factors among the snakebite patients in Somali region, Ethiopia

**DOI:** 10.3389/fpubh.2022.901414

**Published:** 2022-09-27

**Authors:** Ahmed Abdullahi, Nejib Yusuf, Adera Debella, Addis Eyeberu, Alemayehu Deressa, Habtamu Bekele, Indeshaw Ketema, Ibsa Mussa Abdulahi, Fitsum Weldegebreal

**Affiliations:** ^1^Department of Medicine, Denan Health Center, Somalia Region, Jijiga, Ethiopia; ^2^School of Medicine, College of Health and Medical Sciences, Haramaya University, Harar, Ethiopia; ^3^School of Nursing and Midwifery, College of Health and Medical Sciences, Haramaya University, Harar, Ethiopia; ^4^Department of Public Health and Health Policy, School of Public Health, Haramaya University, Harar, Ethiopia; ^5^School of Medical Laboratory Sciences, College of Health and Medical Sciences, Haramaya University, Harar, Ethiopia

**Keywords:** seasonal variation, treatment outcome, snakebites, associated factors, Ethiopia

## Abstract

**Background:**

Snakebite is a major cause of mortality and morbidity in many areas, particularly in the rural tropics, and is a major public health problem around the world. It also imposes significant economic burdens on snakebite victims due to treatment-related expenses and lost productivity.

**Objective:**

The purpose of this study was to assess seasonal variation, treatment outcomes, and its associated factors among snakebite in Denan health center in the Somali region, Ethiopia.

**Method:**

A facility-based cross-sectional study was conducted from 10 to 30 September 2020 in Denan health center, Somali region, Ethiopia. *All snakebite cases in Denan health center from 1 September 2015 to 31 August 2020 were included*. Data were collected using a pre-tested structured checklist from the patient cards. Data were entered into EpiData version 3.1 and analyzed using SPSS version 22 (IBM SPSS Statistics, 2013). The prevalence was reported by proportion with 95% confidence interval (CI) and summary measures. Predictors were assessed using a multivariable logistic regression analysis model and reported using an adjusted odds ratio with 95% CI. Statistical significance was declared at *p*-value < 0. 05.

**Result:**

The overall prevalence of poor outcome of venomous snakebites was 31.4% (95% CI 26.3% 35.4%). Study participants with an age of less than 10 years old (AOR = 2.01; 95% CI 1.39, 4.05), age between 10 and 30 years old (AOR = 2.06; 95% CI 1.39, 9.30), arrival times greater than or equal to 6 hours (AOR = 2.37; 95% CI 1.39, 4.05), and timing of snakebite (AOR = 0.49; 95% CI 0.31–0.87) were factors found to be significantly associated with poor treatment outcome.

**Conclusion:**

According to this study, about one in every three snakebite patients have a poor outcome. Patients with poor outcomes were those who did not improve as a result of treatment or died as a result of it. Designing appropriate engagement of public health education about snakebite prevention techniques, particularly during entry and exit of rainy seasons and establishment of appropriate case management protocol is strongly recommended, as well as increasing the accessibility or availability of antivenoms will undoubtedly have a significant impact on the reduction of mortality and disability related to that of the snakebites.

## Introduction

According to the World Health Organization (WHO), venomous snakes occur in all regions of the globe and are a public health problem, especially in tropical areas. Envenomation due to snakebites is considered one of the main neglected tropical diseases, affecting the poor rural populations of Africa, Asia, Latin America, and Oceania (1).

Snakebite, particularly in the rural tropics, is a major cause of mortality and morbidity, and it has a significant impact on human health and the economy through treatment-related expenditure and loss of productivity (2). Snakebite is the single most important cause of envenoming worldwide and results in substantial mortality in many parts of Africa, Asia, and America (3). Snakebite is significantly neglected as a public health problem in the world as evidenced by the lack of available incidence data from most of the rural tropics where snakebites occur frequently.

Global snakebite (envenoming) incidence has been estimated as 500,000 and mortality between 30000 and 40000 per year (4, 5). Chippaiux estimated that venomous snakes cause 5.4 million bites, approximately 2.5 million envenomings, and over 125,000 deaths worldwide annually (5). White estimated more than three million bites per year resulting in more than 150,000 deaths (6). Details of the methods used to estimate these numbers have not been clearly described. More recently, Anuradhani et al. reported that globally at least 421,000 envenomings occurred annually, but this may be as high as 1,841,000 (7). According to this estimate, the highest numbers of envenoming are estimated for South Asia (121,000), followed by South East Asia (111,000), and East Sub-Saharan Africa (43000).

Health systems in many countries where snakebites are common often lack the infrastructure and resources to collect basic research and statistical data on the problem. Assessing the true impact is further complicated by the fact that cases reported to health ministries by clinics and hospitals are often only a small proportion of the actual burden. This is because many victims never reach primary care facilities and are therefore unreported. This is contributed by socio-economic and cultural factors that influence treatment-seeking behavior with many victims opting for traditional practices rather than hospital care (8).

Venomous snakebites can result in life-threatening medical emergencies. These include significant paralysis, which can cause breathing problems, bleeding disorders, which can result in lethal hemorrhage, irreversible kidney failure, and severe local tissue destruction, which can result in permanent disability and amputation. In many countries, effective treatment for snakebite envenoming is currently unavailable. When treatment is available, the cost can be prohibitively expensive. Long-term consequences of poor treatment can push vulnerable poor people deeper into debt and poverty (9).

According to the Global Burden of Disease 2016 study, the total disability-adjusted life years lost due to venomous animal attacks in West Africa in 2016 is estimated to be around 330,000. An epidemiological survey conducted by the Ethiopian Public Health Institute in 2014 revealed that a total of 949 snakebites were identified over 10 months, with the highest number reported in Oromia, concluding that snakebite is a public issue in Ethiopia (10).

The vicious cycle begins with a lack of appropriate research-based data on actual snakebite epidemiology, which leads to poor local and national health policy regarding snakebite disaster planning, decreased antivenom demand in the market, and finally reduced antivenom production by manufacturers. The vicious cycle continues, and snakes continue to be a neglected tropical disease recognized by WHO (11).

In Ethiopia, available reports and research on snakebite problems are limited, and snakebite is widely regarded as a minor public health concern. However, some small-scale studies, mostly conducted in the northern part of the country, revealed the existence of the problem, with a higher mortality rate of about 14.8%. Furthermore, the experience of professional health workers working in the country's lowland regions reveals significant morbidity and mortality associated with snake accidents, particularly during rainy seasons (12). As a result, the purpose of this study is to assess seasonal variation, treatment outcome, and its associated factors among the snakebite patients in the Somali region, Eastern Ethiopia.

## Methods and materials

### Study design, period, and area

A facility-based cross-sectional study was conducted from 10 to 30 September 2020 in Denan health center, Somali region, eastern Ethiopia. Denan district is geographically located 1123 kilometers (km) southeast of Addis Ababa, 490 km southeast of Jigjiga, and 70 km north of Godey town. Denan woreda has two health centers and nine health posts. The nearest hospitals are Godey hospital (70 km) and Kebridahar Hospital (100 km). The climate in the area is hot, harsh semi-desert weather with less rain full. The total population of Denan woreda are about 33,784 people according to the last Ethiopian national census of 2007. The study was included all snakebite patients registered from 1 September 2015 to 31 August 2020.

### Study population

All snakebite cases in Denan health center from 1 September 2015 to 31 August 2020 were the study population. Since there were small numbers of source population in the study area, all snakebite cases during the study period were included. Patients with no available data or incomplete data registration were excluded.

### Sample size determination and sampling procedure

The sample size was calculated by using a single population proportion formula as follows:


$$\begin{array}{l} n=\left(Z\alpha /2\right)2p\;\left(1-p\right)/d2 \end{array}$$

n = the minimum sample size required, p = estimated proportion of snakebite, and Zα/2 = the value of standard score at 95% confidence interval (1.96), with the following assumptions: Confidence level at 95% = 1.96; margin of error (d) = 0.05, and non-response rate = 10% are considered to get appropriate sample size with available resource, and the proportion of snakebite (50%) was used. The sample size was calculated as (Zα/2) 2^*^ P(1-q) /d2 = (1.96)2 ^*^ 0.5^*^ 0.5/ (0.05)2 = 384 by adding non-response rate (10%), and the final sample size was 422.

Because the total number of admissions at Denan Health Center from 1 September 2015 to 31 August 2020 were 430, all snakebite victims who visited the facility were included.

### Data collection method and procedure

A structured pre-tested extraction sheet adopted from previous similar studies was used to collect data (13–16). The extraction sheet includes socio-demographic characteristics of the study participants, characteristics of snakebite events, clinical presentations, interventions and outcomes of snakebite, and seasonal variation of snakebite. The tool was prepared in the English version. The data extraction was performed by well-trained 10 Bachelor of Science holding Nurses and supervised by two Master of Science-holding Nurses. Before starting data collection, the medical registration number (MRN) of all snakebite patients were traced from the emergency and ward (inpatient) logbooks and by using the list of organized chart numbers, and medical charts were collected from the card room. Then, all necessary information were retrieved from medical charts.

The quality of the data was guaranteed by pretesting using 5% of the respondents who were not eligible for the study before actual data collection. The completeness of the data was checked daily.

### Operational definition

The treatment outcome of snakebite cases that were treated in the health center was categorized as poor (when a patient died or had permanent physical damage or debilitation, such as amputation of a limb or organ failure) or good (an improved health condition).

Antivenom (also called antivenin): the only specific antidote to the toxins in the venom of a particular snake.

Venomous snake: snakes that inject their venoms into humans.

Non-venomous snake: snakes that do not inject venoms.

### Data processing and analysis

The data were entered into Epi data statistical software version 3.1 and then exported to SPSS window version 22 for analysis. Descriptive statistical analysis was used to describe the characteristics of study participants. Then, the information was presented using frequencies, tables, and figures. Binary logistic regression was fitted to see the association between each independent variables and outcome variable. The assumptions of binary logistic regression were checked. The goodness of fit was checked by Hosmer-Lemeshow statistic and omnibus tests. All variables with *P*-value < 0.2 in the bivariate analysis were included in the multivariable analysis to control all possible confounders. The multi-co-linearity test was carried out to see the correlation between independent variables by using the standard error and collinearity statistics (variance inflation factors >10 and standard error >2 were considered as suggestive of the existence of multi-co-linearity). The direction and strength statistical association was measured by odds ratio with 95% CI. Adjusted odds ratio along with 95% CI was estimated to identify the association between independent variables and treatment outcome of snakebite by using multivariable analysis. In this study, *P*-value < 0.05 was considered to declare a statistically significant result.

## Result

### Socio-demographic characteristics

During the 5 years, there were 430 snakebite cases. “Nearly three-fourths [310 (72.1%)] of the study participants were below 25 years.” The majority of the study participants were males [255 (59.3%)], single in marital status [295 (68.6%)], and rural residents [315 (73%)] (Table 1).

**Table 1:** Socio-demographic characteristics of study participants at Denan health center, Shabelle zone, Somali region, Ethiopia.

**Variable**	**Category**	**Frequency (*n* = 430)**	**Percent (%)**
Age	Below 9 years	170	39.5
	10–25 years	140	32.6
	26–40 years	75	17.4
	41–55 years	20	4.7
	>56 years	25	5.8
Gender	Male	255	59.3
	Female	175	40.7
Marital	Single	295	68.6
	Married	135	31.4
Address	Rural	315	73.3
	Urban	115	26.7
Educational Status	Illiterate	95	22.1
	Can read and write	190	44.2
	Primary school	100	23.3
	Secondary school	35	8.1
	College and above	10	2.3
Occupation	Farmer	30	7.0
	Animal herding	60	14.0
	Housewife	65	15.1
	Daily labor	20	4.7
	Government employee	10	2.3
	Merchant	25	5.8
	Others^*^	220	51.2

* Private workers, students.

### Characteristics of snakebite events

Among the study participants, 155 (36%) snakebite cases have occurred during daytime outdoors. Nearly, one-fourth [120 (27.9%)] of the cases the snake attack occurred during sleeping time. The lower extremity was the most common anatomic site for snakebite [190 (44.2%)] followed by the upper extremity [130 (30.2%)]. The majority [350 (81.4%)] of snakebites were venomous bites. Three hundred forty (44.2%) of the study participants arrived at the health institution as an emergency case after 6 hours of the accident. More than half [235 (54.7%)] of the patients were discharged within the first 24 hours of admission (Table 2).

**Table 2:** Characteristics of snakebite among study participants at Denan health center, Shabelle zone, Somali region, Ethiopia.

**Variable**	**Category**	**Frequency(*n* = 430)**	**Percent (%)**
Timing of bite	Day time indoors	85	19.8
	Day time outdoors	155	36.0
	Night time indoors	140	32.6
	Night time outdoors	50	11.6
Place during bite	Snake in the house	155	36.0
	Snake in a ground hole	95	22.1
	Snake in the bush and grassland	150	34.9
	Snake in the garbage around the house	30	7.0
Activities during bite	Walking in the bush	220	51.2
	Cleaning garbage	40	9.3
	Sleeping in the home	120	27.9
	Trying to kill the snake	50	11.6
Affected body parts	Head and neck region	45	10.5
	Upper extremities	130	30.2
	Chest	10	2.3
	Abdomen	15	3.5
	Back and pelvic	25	5.8
	Lower extremities	190	44.2
	Spitting eyes	15	3.5
Characteristics of bite	Dry bite	65	15.1
	Non-dry bite	350	81.4
	Spitting in the eyes of the victim	15	3.5
Arrival time at the health facility	Within 1 h	30	7.0
	2–3 h	20	4.7
	3–4 h	10	2.3
	4–6 h	30	7.0
	>6 h	340	44.2
Health facility stays	Less than one day	235	54.7
	1–5 days	100	23.3
	6–10 days	55	12.8
	11–15 days	10	2.3
	>15 days	30	7.0

### Clinical presentations, interventions, and outcomes of snakebite

The most common clinical manifestations in snakebite patients were localized symptoms such as pain (24.4%), swelling (31.4%), and soft tissue necrosis (29.1%) around the area of snakebite. Among study participants, 380 (88.4%) did not have clinical features of systemic envenomation with variable severity range. More than one-third [160 (37.3%)] of study participants had any traditional remedies before presenting to the health center (Table 3).

**Table 3:** Clinical presentations, interventions, and outcomes of snakebite among study participants at Denan health center, Shabelle zone, Somali region, Ethiopia.

**Variables**	**Categories**	**Frequency**	**Percentage**
Clinical presentations	Only fang marks	65	15.1
	Local pain	105	24.4
	Local swelling	135	31.4
	Local tissue necrosis	125	29.1
	Neurologic disorders	15	3.5
	Bleeding disorder	15	3.5
	Anaphylactic shock and hemodynamic instability	20	4.7
	No systemic envenomation	380	88.4
Traditional medication used	Herbal medication	100	23.3
	Tourniquet applied	60	14.0
	None	270	62.8
Treatment provided	Analgesia-only	220	51.2
	Analgesia + IV fluid (colloid)	75	17.4
	Daily wound care + wound debridement + TAT, antibiotics	135	31.4
Treatment outcome	Improved and discharged	295	68.6
	Referred to higher hospital	20	4.7
	Local tissue loss	90	20.9
	Limb amputation	5	1.2
	Death	20	4.7
Referral	For mechanical ventilation	5	1.2
	For amputation	10	2.3
	For blood transfusion	10	2.3
	For fasciotomy	5	1.2
	None	390	90.7
	Renal failure	10	2.3

IV = Intravenous; TAT = Tetanus antitoxin.

Most of patients were given supportive care including pain and fluid management, with local wound care and antibiotics. There was no registration of any patient received antivenom in the health center. The overall prevalence of poor outcome of venomous snakebites was 31.4% [95% CI 26.3% 35.4%]. Among study participants, 4.7% of study participants died in a health center due to a snakebite. Regarding major clinical manifestations, bleeding (2.3%), and amputation (2.3%) were the most common (Table 3).

### Seasonal variation of snakebite

**Figure 1:**
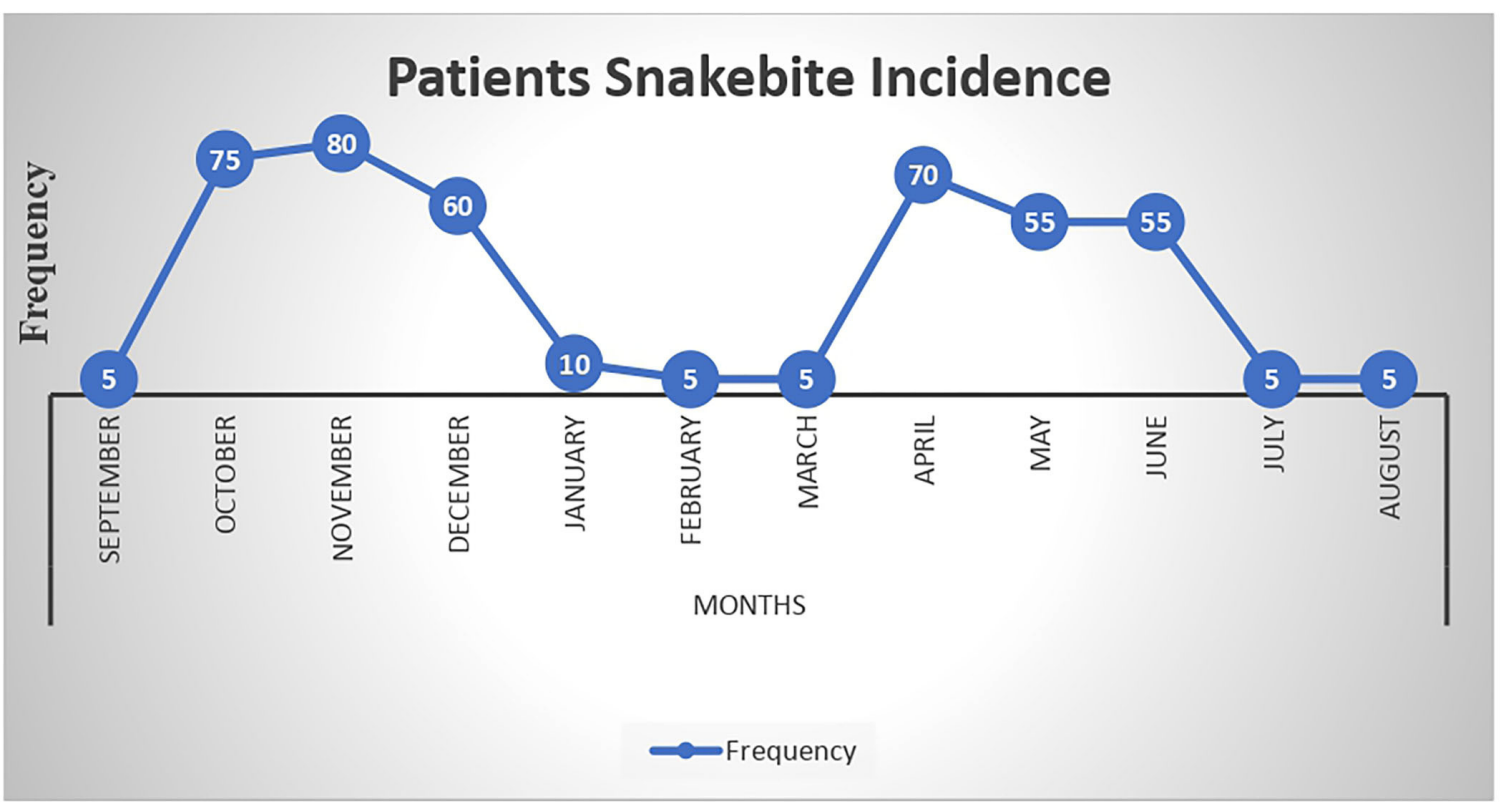
Monthly seasonal variation of snakebite among study participants at Denan health center, Shabelle zone Somali region, Ethiopia.

**Figure 2:**
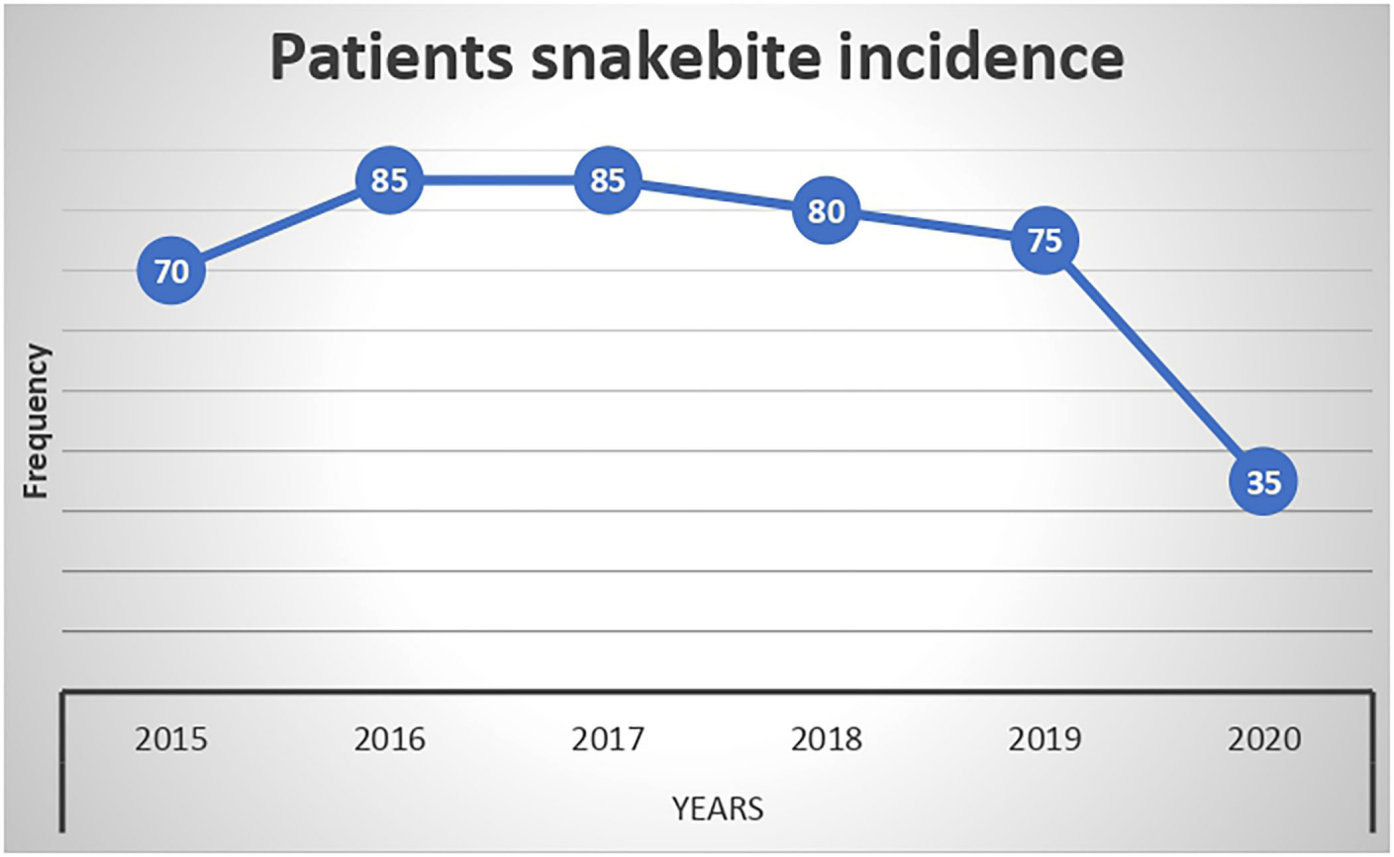
Yearly variation of snakebite among study participants at Denan health center, Shabelle zone Somali region, Ethiopia.

The entry and exit of rainy seasons of the year were associated with an increase in the frequency of snakebite, with April to June and October to December having the highest snakebite occurrence (Figure 1). The frequency of snakebite decreased from 2016 to 2020 (Figure 2).

### Factors associated with snakebite outcome

In the final multivariable logistic regression model, variables such as the age of patient, residency, arrival time, and timing of snakebite were significantly associated with poor outcomes. The odds of having poor outcomes among study participants aged below 10 years and 10–30 years were 4.63 times ([AOR = 4.63, 95% CI (2.13–11.38)] and 2.42 times [AOR = 2.42, 95% CI (1.39–9.30)]) higher than those participants aged above 30 years. Urban residents had 44% reduction [AOR = 0.56, 95% CI (0.06–0.97)] of poor outcomes than those study participants residing in rural. Those participants who had suffered the snakebite at daytime were 51% reduced [AOR = 0.49, 95% CI (0.31–0.87)] to have poor outcome compared to those who suffered snakebite at night time. Those patients who arrived late at the health facility for more than 6 hours were 2.01 times [AOR = 2.01, 95% CI (1.39–4.05)] more likely to have poor outcomes than their counterparts (Table 4).

**Table 4:** Factors associated with outcome among snakebite patients attending Denan health center, Ethiopia, 2020.

**Variable**	**Outcome**		**COR 95% CI**	**AOR 95% CI**	***P*-value**
	**Poor**	**Good**			
**Residence**					
Urban	35 (30.4%)	80 (69.6%)	0.94 (0.14–0.99)	0.56 (0.06–0.97)	0.01
Rural	100 (31.7%)	215 (68.3%)	1	1	
**Sex**					
Male	90 (35.3%)	165 (64.7%)	1.58 (1.01–2.90)	1.56 (1.21–3.44)	0.228
Female	45 (25.7%)	130 (74.3%)	1	1	
**Age**					
Less than 10 years	80 (47.1%)	90 (52.9%)	9.7 (4.78–19.96)	4.63 (2.13–11.38)	0.001^*^
10–30 yrs	45 (32.1%)	95 (67.9%)	5.21 (1.36–8.72)	2.42 (1.39–9.30)	0.001^*^
Above 30 yrs	10 (8.3%)	110 (91.7%)	1	1	
Educational status					
Cannot read and write	55 (57.9%)	40 (42.1%)	1.15 (1.01–6.81)	2.01 (0.75–5.44)	0.168
Primary school	60 (31.6%)	130 (68.4%)	1.67 (1.37–3.64)	1.50 (0.26–3.13)	0.873
Secondary school	15 (30.0%)	35 (70.0%)	0.35 (0.02–3.42)	1.84 (0.87–9.30)	0.084
College and above	6 (54.5%)	5 (45.5%)	1	1	
**Timing of snakebite**					
Day time	65 (27.1%)	175 (72.9%)	0.637 (0.04–0.96)	0.49 (0.31–0.87)	0.02^*^
Nighttime	70 (36.8%)	120 (63.2%)	1	1	
**Arrival time**					
6 hours and above	40 (44.4%)	50 (55.6%)	2.06 (1.24–3.33)	2.01 (1.39–4.05)	0.001^*^
Less than 6 hours	95 (27.9%)	245 (72.1%)	1	1	

## Discussion

This study assessed the clinical presentations, interventions, treatment outcomes of snakebites, and their associated factors in the Denan health center in the Somali region, Eastern Ethiopia. It revealed that 4.7% of them have died in the health center due to snakebite from 1 September 2015 to 31 August, 2020. Moreover, this study pointed out that bleeding (3.5%), amputation (2.3%), and renal failure (2.3%) were the most common clinical manifestations. Age of patient, residency, arrival time, and timing of snakebite were identified as predictors of poor treatment outcome.

In this study, the poor treatment outcome among the snakebite patients was 31.4% [95% CI 26.3% 35.4%]. It also showed that 20 (4.7%) of the 430 patients who were bitten by a snake died. This is consistent with the study conducted in Nepal (17) and Zimbabwe (18). Taking into consideration the population that resides in the catchment area and uses Denan health center for health services, one possibly says that death from the snakebites was high. This is much higher than the study conducted in the most developed country while much lower than the study conducted in developing country (5). This high death rate could be attributed to a lack of antivenom and late arrival at the health center, both of which play a critical role in the death of snakebite patients (19, 20).

In this study, monthly admissions to the health center showed a clear seasonal trend between 2015 and 2020, with the majority of patients admitted between April and June and October and December. In Ethiopia, it is crystal clear that these months, such as April and June, are a rainy season during which labor-intensive agricultural activities such as land preparation for planting and sowing, as well as weeding, are carried out. To that end, an increased number of workers will be exposed to the field, potentially making them vulnerable to snakebites. This is in harmony with the study conducted in California (21), Costa Rica (22), and Brazil (23). This is due to the fact that the start of the rainy season coincides with an increase in the number of accidents, a situation that has been reported in other tropical regions (24–26). Furthermore, the rainy season coincides with the birth season of several snake species, which contributes to an increase in the number of snakebites (27). Similarly, this study found an increase in snakebite accidents during the months of October and November. This finding is supported by studies conducted in Brazil (23) and California (21). One possible explanation is that this is a month when there is a lot of harvesting going on, which exposes workers to snakebites.

According to this study, the odds of having poor outcomes among study participants aged below 10 years and 10–30 years were 4.63 and 2.42 times higher than those participants aged above 30 years ([AOR = 4.63, 95% CI (2.13–11.38)] and [AOR = 2.42, 95% CI (1.39–9.30)]), respectively. This is in line with a study conducted in Costa Rica (22). One possible explanation would be that young people are more exposed to snakebite due to their greater activity in the field. Another possible reason could be because of the relative frequency of each age group in the population. Furthermore, if snakebites occur in children, the likelihood of a poor treatment outcome increases. This could be related to the fact that as people get older, their level of caution increases, which plays a critical role in their ability to avoid being harmed by snakes. Furthermore, bites in children are more complicated than bites in adults, presumably owing to their lower body mass and faster absorption (28).

In this study, those who were urban residents had a 44% reduction of poor outcomes than those study participants residing in rural [AOR = 0.56, 95% CI (0.06–0.97)]. This is in line with the study conducted in Northwest Ethiopia (29). One possible reason is that they are more likely to arrive at the health center quickly because they are in a nearby health center, as opposed to those who come from a remote area and thus receive timely care and management. Furthermore, those who live in urban are more likely to accept treatment-related advice than those who live in rural areas, which may have a positive effect on treatment outcomes.

According to the findings of this study, those who were bitten by a snake during the day had a 51% lower chance of having a poor outcome than those who were bitten at night [AOR = 0.49, 95% CI (0.31–0.87)]. The possibility is that the likelihood of receiving treatment will increase because means of transportation are more easily available during the day, increasing the likelihood of arriving at a health center where treatment is provided. Furthermore, the depth and intensity of bites at night may be severe, as darkness may prevent victims from being protected from snakebites.

The finding from this study revealed that those patients who arrived late at the health facility after more than 6 hours were 2.01 times more likely to have poor outcomes than their counterparts [AOR 2.01, 95% CI (1.39–4.05)]. This is in line with the study conducted in Brazil (23). This is because the time between treatment and initiation of medical care is directly correlated. Thus, time to treatment variables is related to the severity of snakebite envenomation.

## Conclusion

According to the study, about one in every three snakebite patients have a poor outcome. Designing appropriate engagement of public health education about snakebite prevention techniques, particularly during the entry and exit of rainy seasons and establishment of appropriate case management protocol is strongly recommended, as well as increasing the accessibility or availability of antivenoms, will undoubtedly have a significant impact on the reduction of mortality and disability related to that of the snakebites.

## Data availability statement

The raw data supporting the conclusions of this article will be made available by the authors, without undue reservation.

## Ethics statement

The studies involving human participants were reviewed and approved by Haramaya University College of Health and Medical Sciences, Institutional Health Research Ethics Review Committee (IHRERC). The participants provided their written informed consent to participate in this study.

## Author contributions

All authors made a significant contribution to the work reported, whether that is in the conception, study design, execution, acquisition of data, analysis, and interpretation, or in all these areas, took part in drafting, revising, or critically reviewing the article, gave final approval of the version to be published, have agreed on the journal to which the article has been submitted, and agree to be accountable for all aspects of the work.

## Funding

Haramaya University provided financial support for this study. However, the funding agency had no role in the collection, analysis, and interpretation of the data as well as the writing of the manuscript.

## Conflict of interest

The authors declare that the research was conducted in the absence of any commercial or financial relationships that could be construed as a potential conflict of interest.

## Publisher's note

All claims expressed in this article are solely those of the authors and do not necessarily represent those of their affiliated organizations, or those of the publisher, the editors and the reviewers. Any product that may be evaluated in this article, or claim that may be made by its manufacturer, is not guaranteed or endorsed by the publisher.
